# Enhanced Cell Growth of Adipocyte-Derived Mesenchymal Stem Cells Using Chemically-Defined Serum-Free Media

**DOI:** 10.3390/ijms18081779

**Published:** 2017-08-16

**Authors:** Myung-Suk Lee, Christine Youn, Jeong Hyun Kim, Byoung Jun Park, Jongchan Ahn, Sungyoul Hong, Young-Deug Kim, Young Kee Shin, Sang Gyu Park

**Affiliations:** 1 Xcell Therapeutics, Hanhwa Biz Metro Building, 242 Digital-ro, Guro-gu, Seoul 152-733, Korea; mslee@xcell.co.kr (M.-S.L.); acadekjh@xcell.co.kr (J.H.K.); bioart96@xcell.co.kr (J.A.); 2 College of Pharmacy, Seoul National University, Kwanak-ro, Kwanak-gu, Seoul 151-742, Korea; christineyoun91@gmail.com; 3 ABION Inc., Hanhwa Biz Metro Building, 242 Digital-ro, Guro-gu, Seoul 152-733, Korea; bjpark@xcell.co.kr (B.J.P.); todnos@abionbio.com (Y.-D.K.); 4 Research Institute of Pharmaceutical Sciences, Seoul National University, Kwanak-ro, Kwanak-gu, Seoul 151-742, Korea; sungyoul@snu.ac.kr; 5 Molecular Medicine and Biopharmaceutical Sciences, Graduate School of Convergence Science and Technology, Seoul National University, Seoul 08826, Korea; 6 Department of Pharmacy, College of Pharmacy, Ajou University, Worldcup-ro, 206, Yeongtong-gu, Suwon 16499, Korea

**Keywords:** mesenchymal stem cell, multipotency, chemically-defined medium, proliferation, differentiation, secretome

## Abstract

The multipotency and anti-inflammatory effects of mesenchymal stem cells (MSCs) make them attractive for cell therapy in regenerative medicine. A large number of MSCs is required for efficient therapy owing to the low homing efficiency of MSCs to target sites. Furthermore, owing to limitations in obtaining sufficient amounts of MSCs, in vitro expansion of MSCs that preserves their differentiation and proliferative potential is essential. The animal factor included in culture media also limits clinical application. In this study, adipose-derived MSCs showed a significantly higher proliferation rate in STK2, a chemically-defined medium, than in DMEM/FBS. The expression of MSC surface markers was increased in the culture using STK2 compared to that using DMEM/FBS. Tri-lineage differentiation analyses showed that MSCs cultured in STK2 were superior to those cultured in DMEM/FBS. In addition, MSCs cultured in STK2 showed a reduced senescence rate, small and homogenous cell size, and were more genetically stable compared to those cultured in DMEM/FBS. Furthermore, secretome analysis showed that the expression of factors related to proliferation/migration, anti-inflammation, and differentiation were increased in STK2 culture medium compared to DMEM/FBS. Taken together, these results suggest that culture using STK2 medium offers many advantages through which it is possible to obtain safer, superior, and larger numbers of MSCs.

## 1. Introduction

Mesenchymal stem cells (MSCs) are stromal multipotent stem cells that can differentiate into a variety of cell types such as adipocytes, chondrocytes, and osteocytes [1,2]. MSCs have been isolated from diverse tissues including fetal liver, umbilical cord blood, bone marrow, hair follicle, and adipose tissue [3]. MSCs are attractive for stem cell therapy because of their ability to differentiate into many cell types and secrete growth factors [4,5,6]. In addition, due to its anti-inflammatory effect, the complications associated with immune rejection of allogeneic tissue can be avoided [7]. Adipose-derived stem cells (ASCs) are one of the most promising MSC populations because they can be easily isolated in large quantities. However, as multiple injections of ASCs are required to treat disease, large numbers of ASCs are required. Therefore, in vitro expansion of ASCs without affecting their differentiation and proliferative potential is essential. Currently, there are limitations to in vitro manipulation of MSCs using fetal bovine serum (FBS) for clinical therapy because of the inclusion of animal-derived factors. However, use of FBS is still allowed, and it is used for in vitro isolation and propagation of MSCs before clinical application. FBS could activate the immune system of a patient upon repeated administration, and increases the risk of transmitting zoonotic viral or prion diseases [8,9,10,11]. In addition, there is no consistency in quality of FBS between lots or batches [12,13]. The levels of growth factors and the plethora of other biomolecules in blood are dependent on their environment, which leads to difficulty in cell culture control. Therefore, the use of FBS is especially inadequate for culture of stem cells for clinical application. The uncharacterized factors in FBS could affect the differentiation potential of cells when administered in vivo [14]. As the clinical risks of MSCs due to the use of FBS have become more recognized, culture media containing a variety of growth factors that could replace animal serum, such as human-serum, platelet lysate derivatives, and sphingosine-1-phosphate have been developed [15,16,17]. Of note, cytokines, including transforming growth factor (TGF-β1), platelet-derived growth factor (PDGF)-BB, PDGF-AB, heparin binding epidermal growth factor (HB-EGF), and insulin growth factor (IGF)-1 are known to mediate proliferation, migration, and maintenance of stemness in MSCs [18,19]. Based on these studies, a variety of molecules, including epidermal growth factor (EGF), basic FGF (bFGF), glucose, cytokines, vitamins, amino acids, fatty acid, and sodium bicarbonate were combined and optimized for in vitro manipulation of MSCs without FBS, which has led to development of STK2 medium [20,21,22,23,24].

In this study, we show that STK2, a chemically-defined serum-free medium, has the following advantages in ASC culture—cell proliferation, population doubling time (PDT), cell viability, differentiation potential, cell population homogeneity, reduced senescence, decreased genetic instability, and secretion of growth factors and anti-inflammatory cytokines.

## 2. Results

### 2.1. Comparison of Cell Growth, Population Doubling Time, and Viability

**Figure 1:**
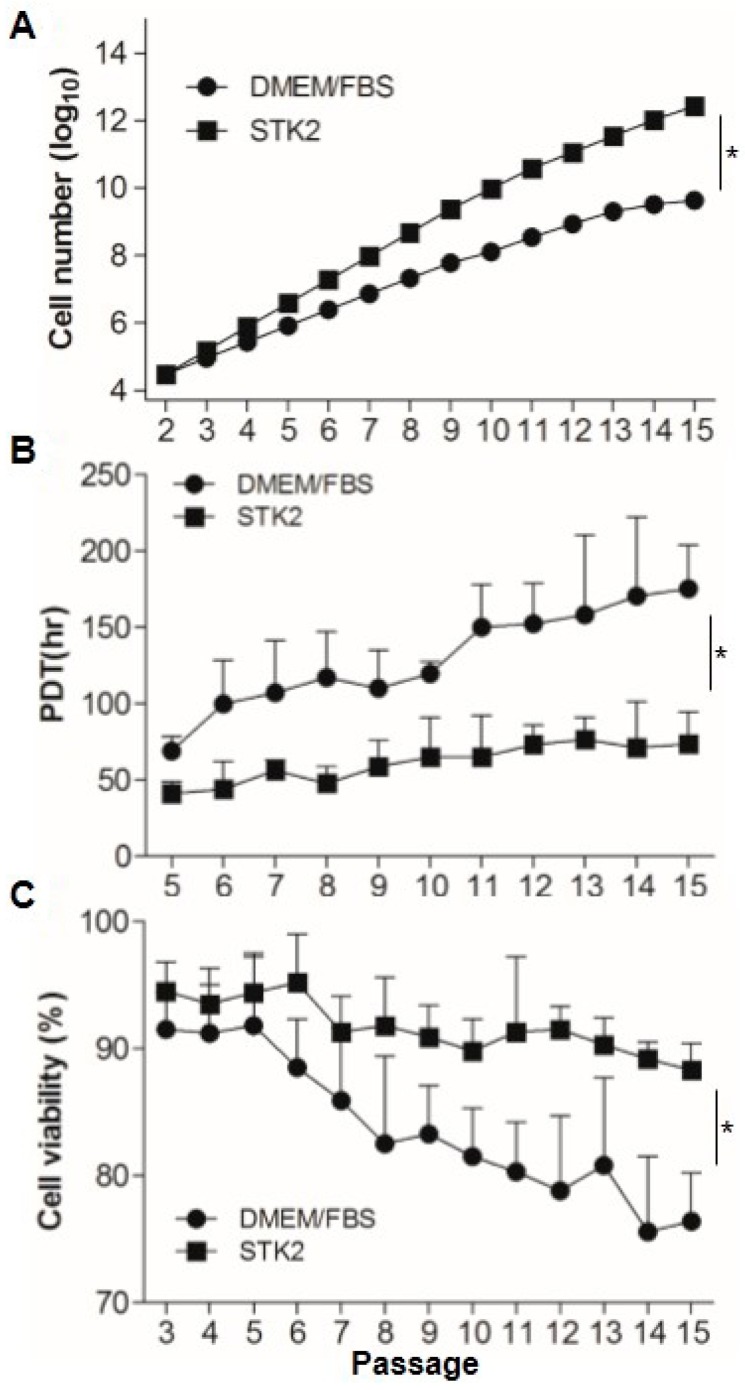
Comparison of cell growth, population doubling time (PDT), and viability of ASCs cultured in DMEM/FBS and STK2. (**A**–**C**) ASCs (3 × 10^4^ cells) at P2 were seeded onto 6 well-plates. Cell numbers and viability were evaluated as described in the Methods section (*n* = 4). PDT was calculated by the following formula: PDT = $T\times \frac{\log\left(2\right)}{\log\left(q2\right)-\log\left(q1\right)}$ (T, culture time; q1, initial number of cells; q2, final number of cells) (*n* = 4). The values are means ± SD values. * *p* < 0.01.

First, we compared the accumulated cell number in the culture using DMEM containing 10% FBS or STK2. Adipose-derived stem cells ASCs (3 × 10^4^ cells) at P2 were seeded onto 6-well plates, and were continuously subcultured to P15. At passage 15, accumulated cell numbers in culture using STK2 were about 2.61 × 10^12^, which exhibited a significant increase compared to that in culture using DMEM containing 10% FBS (4.28 × 10^9^) (Figure 1A). It is known that maintenance of a steady PDT is critical for amplification of MSCs to treat diseases. The culture using STK2 maintained a significantly stable PDT between 1.60 and 1.82 from P5 to P15, whereas PDT of the culture using DMEM containing 10% FBS increased to 2.1 at P6, then 2.4 at P15, suggesting that the culture using STK2 could harvest more cells in a faster time compared to DMEM containing 10% FBS (Figure 1B). In addition, the culture using DMEM containing 10% FBS showed that cell viability from P3 to P5 was maintained at about 93%, then gradually decreased to about 75% at P15. Interestingly, cell viability in the culture using STK2 was stably maintained at about 90% to P15. Taken together, these results show that the culture of ASCs using STK2 offers many advantages in terms of cell number, viability, and culture time.

### 2.2. Comparison of Biomarker Expression

**Figure 2:**
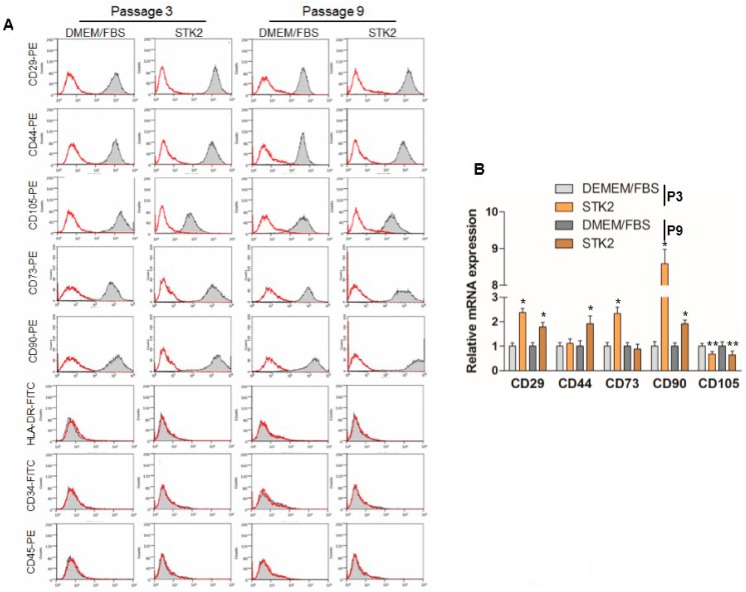
Analysis of ASC marker expression. (**A**) ASCs were cultured in DMEM/FBS or STK2, and stained with anti-CD29-PE, anti-CD44-PE, anti-CD73-PE, anti-CD90-PE, and anti-CD105-PE antibodies as positive markers, and anti-HLA-DR-FITC, -CD34-FITC, and -CD45-PE antibodies as negative markers. A representative image from three independent experiments is shown; (**B**) Total RNAs were isolated and qRT-PCR was performed to analyze the expression of CD markers as described in the Methods section. Data represent the mean ± SEM as an average of three independent experiments. * and ** vs. corresponding passage DMEM/FBS. * *p* < 0.01; ** *p* < 0.05.

The expression of ASC surface markers, including CD29, CD44, and CD105, was examined by using FACS analysis to compare ASCs cultured in DMEM/FBS with those cultured in STK2. The cultured ASCs were shown to be positive for CD29, CD44, CD73, CD90, and CD105, but negative for CD34, CD45, and HLA-DR in both DMEM/FBS and STK2 (Figure 2A). Interestingly, the expression levels of CD29, CD44, CD73, and CD90 of ASCs cultured in STK2 were higher compared to those cultured in DMEM/FBS in both FACS and qRT-PCR analyses (Table 1, Figure 2A,B). However, the ASC expression level of CD105 in STK2 culture was shown to be lower than that in DMEM/FBS in both FACS and qRT-PCR analyses (Table 1, Figure 2A,B). It is known that culture using serum-free media leads to reduced expression of CD105 [25]. Although CD105^+^ MSCs are known to be superior to unselected MSCs in regeneration of post-infarction heart [26,27], the effect of reduced expression of CD105 in culture using STK2 on therapeutic efficacy needs further investigation.

### 2.3. Differentiation Analysis

**Figure 3:**
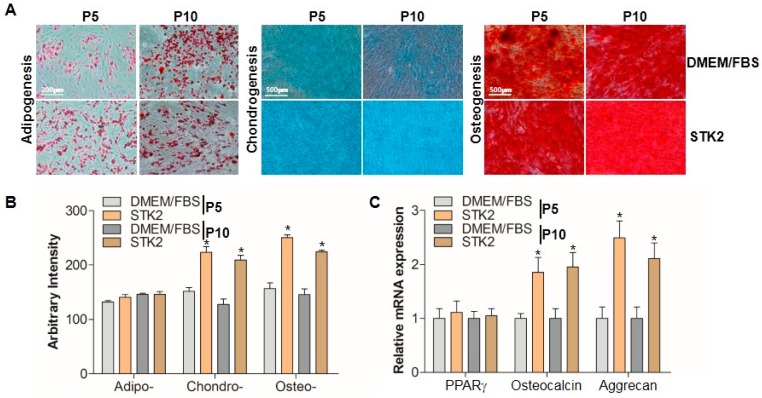
Comparison of trilineage differentiation capability of ASCs cultured in DMEM/FBS and STK2. (**A**) Differentiation into adipocytes, chondrocytes, and osteocytes was induced and stained as described in the Methods section. A representative image from three independent experiments is shown; (**B**) The staining intensity was quantitated and evaluated by ImageJ, and expressed as arbitrary intensity; (**C**) The mRNA expression levels of PPARγ (adipogenic marker), osteocalcin (osteogenic marker), and aggrecan (chondrogenic marker) were quantitated using qRT-PCR and presented as relative expression (*n* = 3). Data represent the mean ± SEM as an average of three independent experiments. * vs. corresponding passage DMEM/FBS. * *p* < 0.05.

It is known that MSCs cultivated ex vivo are able to differentiate into three separate mesenchymal lineages [28]. To examine whether differentiation capability would be affected by serum-free conditions, ASCs were cultured in DMEM/FBS and in STK2 medium, and stimulated to commit to one of three lineages. At the end of differentiation, cells were stained as described in the Methods section, and imaged using a phase-contrast microscope (Figure 3A). Adipogenic differentiation was determined by observing the presence of Oil Red O-stained fat vacuoles in cells (Figure 3A). Chondrogenic differentiation was evaluated by Alcian Blue staining in regions saturated with extracellular matrix composed of acidic polysaccharides that are highly expressed in the cartilage (Figure 3A). Similarly, osteogenic differentiation capacity was determined by Alizarin Red S staining, which marked differentiated calcium-rich extracellular matrix regions (Figure 3A). Both DMEM/FBS and STK2 groups showed trilineage differentiation capabilities. Densitometric analysis showed that adipogenic differentiation capability was the same in DMEM/FBS and STK2 groups (Figure 3B). Interestingly, the chondrogenic and osteogenic differentiation capabilities of ASCs cultured in STK2 were significantly higher than those cultured in DMEM/FBS (Figure 3B). The expression levels of each differentiation marker, including PPARγ (adipogenesis), osteocalcin (osteogenesis), and aggrecan (chondrogenesis) were examined using quantitative RT-PCR analysis as described previously [29]. Unlike PPARγ, mRNA expression levels of osteocalcin and aggrecan were significantly higher in ASCs cultured in STK2 compared to those cultured in DMEM/FBS (Figure 3C). These results further supported that ASCs cultured in STK2 have stronger capabilities to differentiate into chondrocytes and osteocytes than those cultured in DMEM/FBS.

### 2.4. Senescence Analysis

**Figure 4:**
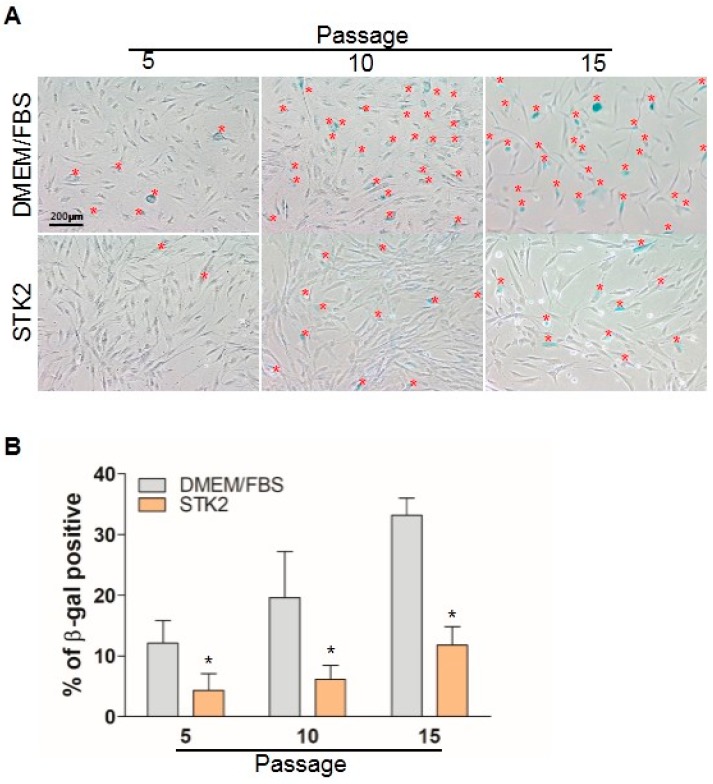
Comparison of senescence of ASCs cultured in DMEM/FBS and STK2. Cells at each passage were seeded and cultured for 24 h, then β-galactosidase was stained as described in the Methods section. (**A**) A representative image from three independent experiments is shown. Red * indicates β-galactosidase positive cell; (**B**) β-galactosidase-positive cells were counted and presented as a percentage. Data are represented as the mean ± SEM of the average of at least three independent experiments. * vs. DMEM/FBS. * *p* < 0.05.

Although MSCs are a promising therapeutic tool for various diseases, MSCs should be expanded through in vitro culture to obtain a sufficient amount of cells prior to clinical application. However, it has been known that continuous subculture induces morphological abnormalities, cell enlargement, and ultimately proliferation arrest known as senescence [30]. Therefore, we compared senescence levels between ASCs cultured in DMEM/FBS and STK2 media. As shown in Figure 4, about 12% of ASCs (12.1 ± 3.7%) at P5 when cultured in DMEM/FBS were shown to be positive for β-galactosidase, a senescence marker, and the β-galactosidase-positive cell population was increased at P10 (19.6 ± 7.6%) and at P15 (33.2 ± 2.8%). Interestingly, the β-galactosidase-positive population of ASCs cultured in STK2 was 4.5 ± 2.7% at P5, 6.2 ± 2.3% at P10, and 11.2 ± 3.1% at P15. These results indicate that culture using STK2 medium has an advantage in reducing cellular senescence of ASCs compared to DMEM/FBS.

### 2.5. Phenotype Analysis

**Figure 5:**
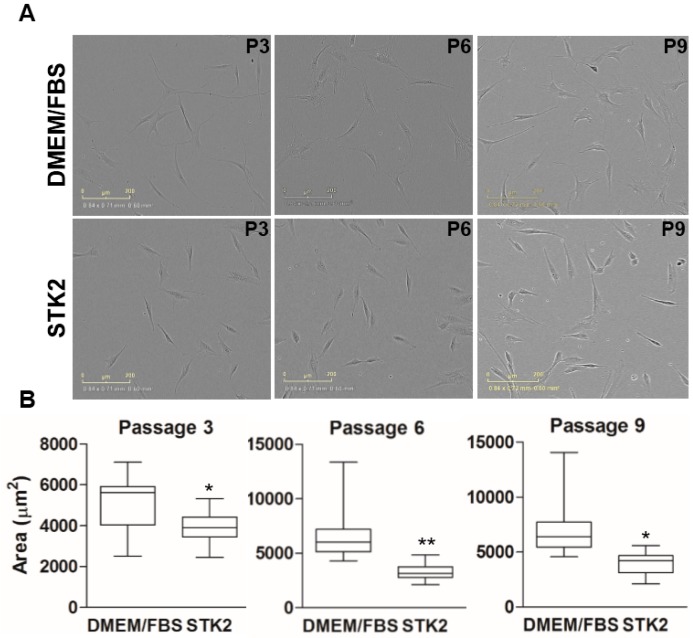
Comparison of cell size of ASCs cultured in DMEM/FBS and STK2. (**A**) Images were captured at the indicated passage using an IncuCyte ZOOM Live-Cell Analysis System at 100× magnification; (**B**) Adherent cell size was measured by analyzing phase-contrast images with ImageJ. Analyzed adherent cell sizes were compared by box-and-whisker plot. Data are represented as the mean ± SD of the average of at least three independent experiments. * and ** vs. DMEM/FBS. * *p* < 0.001; ** *p* < 0.0001.

ASCs isolated from human adipose aspirates in either DMEM/FBS or STK2 exhibited slightly differing adherent morphologies. Cells in both groups showed fibroblastic morphology, but ASCs cultured in DMEM/FBS showed longer elongation of branches, and correspondingly, larger adherent sizes compared to those cultured in STK2 (Figure 5A). The morphologies across passages showed increased variation when cultured in DMEM/FBS compared to STK2 (Figure 5B). ASCs cultured in STK2 continued to show fibroblastic morphology with slight cell body enlargement, whereas ASCs cultured in DMEM/FBS showed a significant increase in cell body width, which resembled the morphology of senescent MSCs, caused by prolonged in vitro culture as previously described [31]. This result is in good agreement with the notion that the culture using DMEM/FBS results in a greater increase in senescence than STK2, as shown in Figure 5. It is known that the most convenient method of MSC transplantation is via intravenous injection. However, systemic intravenous injection can result in MSCs residing in lung, liver, spleen, intestine, and bone marrow [32,33]. Lung entrapment of MSCs, particularly, may cause unwanted side effects such as embolism in small capillaries. For this reason, cell size should be small when systemically administered.

**Figure 6:**
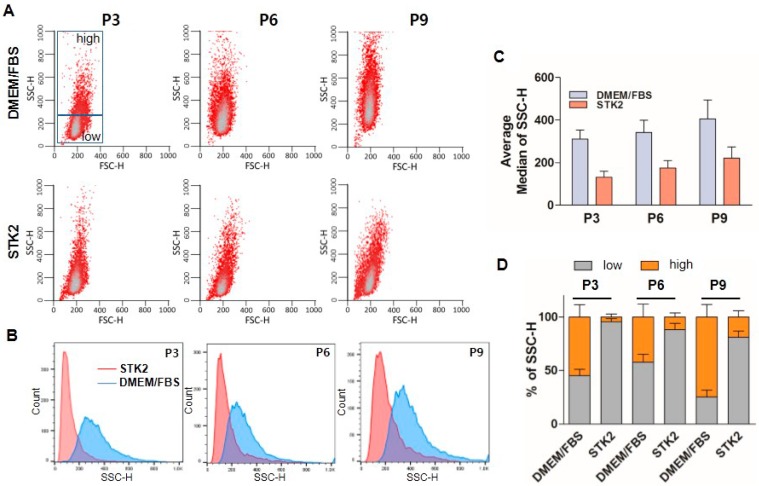
FACS analysis of ASCs cultured in DMEM/FBS and STK2. (**A**) Cellular homogeneity of ASCs was analyzed by FACS; (**B**) SSC-H distribution; and (**C**) average median values of SSC-H were compared using each indicated passage of ASCs cultured in DMEM/FBS and STK2; (**D**) Values over 300 in SSC-H were grouped as the high SSC (HSSC) population and values below 300 in SSC-H were grouped as the low SSC (LSSC) population. The change of each population is presented as a bar graph (*n* = 3).

Side scatter channel (SSC) is a measure of the cell refractive index, dependent on cell granularity or internal complexity, including cytoplasmic membrane wrinkling, number and shape of vesicles and mitochondria, development of endoplasmic reticulum, and nucleus structure [34]. FACS analysis showed that ASCs cultured in DMEM/FBS were more heterogeneous compared to those in STK2, and SSC values were increased and varied with consecutive culture across passages in the group of ASCs cultured using DMEM/FBS. On the other hand, ASCs cultured using STK2 maintained a relatively stable SSC population (Figure 6A, B). SSC average median values of ASCs cultured in DMEM/FBS were 311 ± 42 (P3), 342 ± 57 (P6), and 406 ± 89 (P9), whereas those of ASCs cultured in STK2 were 131 ± 28 (P3), 175 ± 35 (P6), and 221 ± 53 (P9), further confirming that ASCs cultured in STK2 were more homogenous compared to those cultured in DMEM/FBS (Figure 6C). In addition, we divided SSC results into two groups, high SSC (HSSC) and low SSC (LSSC), based on values above or below 300, respectively, as shown in Figure 6A. The HSSC population of ASCs cultured in DMEM/FBS was dramatically higher than that cultured in STK2 (Figure 6D). It is known that HSSC populations show increased apoptosis and aneuploidy compared to LSSC [34], suggesting that ASCs cultured in STK2 might be healthier and more genetically stable compared to those cultured in DMEM/FBS. Taken together, these results suggest that culture of ASCs using STK2 has an advantage in the harvest of a healthy, small size, and homogenous cell population.

### 2.6. Comparison of Genetic Stability

**Figure 7:**
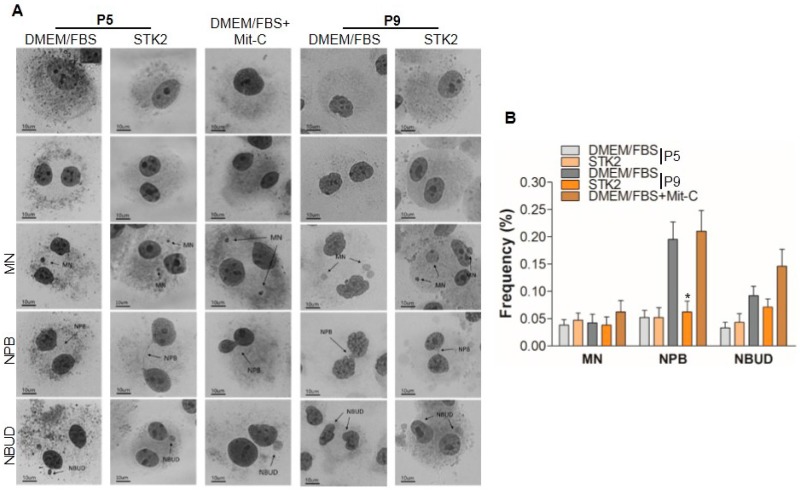
Analysis of genetic stability of ASCs cultured in DMEM/FBS and STK2. To examine the genetic stability of ASCs, cells were cultured in the presence or absence of mitomycin C (mit-C), and were subjected to CBMN assay as described in the Methods section. Mit-C was used as the positive control. (**A**) Binucleated cells were analyzed for the presence of micronuclei (MN), nucleoplasmic bridges (NPB), and nuclear buds (NBUD). Images were captured at 400× (scale bar, 10 μm); (**B**) Each frequency was evaluated and presented as a bar graph (*n* = 4). Data represent the mean ± SD of the average of at least three independent experiments. * vs. DMEM/FBS (P9). * *p* < 0.001.

Although a number of studies have shown that stem cells remain morphologically and genetically stable after in vitro culture [35,36], we cannot rule out the possibility of genetic instability during cell expansion because transformation of MSCs, even of a single cell, can induce a variety of cancers [37,38,39,40]. Therefore, we examined and compared the genetic stability of cells cultured in DMEM/FBS and STK2 using cytokinesis-block micronucleus (CBMN) assay. Nuclear alterations in micronuclei (MN), nucleoplasmic bridges (NPBs), and nuclear buds (NBUDs) of binucleated cells were examined and evaluated using images of Giemsa-stained cells (Figure 7). There was no difference in the formation of MN and NBUD between ASCs cultured in DMEM/FBS and STK2 (Figure 7A). However, ASCs cultured in DMEM/FBS at P9 showed a significant increase in the formation frequency of NPBs comparable to the mit-C treated group (positive control), whereas ASCs cultured in STK2 at P9 did not show any differences compared with P5, suggesting that culture using STK2 is more genetically stable compared to DMEM/FBS (Figure 7B). NPBs indicate the occurrence of rearrangements in which chromatids or chromosomes (including dicentric chromosomes and ring chromosomes) are pulled to opposite poles during anaphase [41]. Since individual MSCs show a broad range of variation, there might possibly be genetic variation in even a small population. Therefore, to reduce genetic instability and for safe clinical application of MSCs, CBMN analysis should be performed even on healthy cultures of MSCs with high viability and proliferation rates.

### 2.7. Secretome Analysis

**Figure 8:**
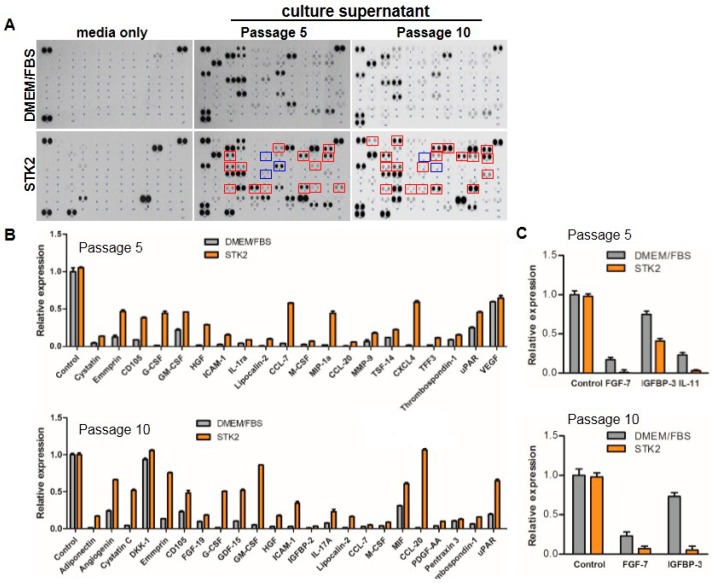
Secretome analysis of ASCs cultured in DMEM/FBS and STK2. (**A**) Supernatant of ASCs cultured in DMEM/FBS or STK2 at the indicated passage was analyzed using the human cytokine proteome array; (**B** and **C**) The relative intensity of each dot corresponding to a different factor was normalized by the intensity of the control. The factors with noticeably different relative detection levels between DMEM/FBS and STK2 groups are presented as bar graphs (*n* = 3).

A number of studies have shown that MSCs secrete a variety of biologically active cytokines/growth factors, extracellular matrix proteins, and tissue remodeling enzymes [42,43]. The various factors secreted by MSCs can suppress local inflammation/immune responses, and reduce oxidative stress, fibrosis, and cell death. To analyze and compare secretomes of ASCs cultured in STK2 and DMEM/FBS, media or culture supernatants were subjected to a cytokine proteome array as described in the Methods section. As shown in Figure 8 and Table 2, factors related to cell proliferation, including angiogenin, cystatin C, G-CSF, GM-CSF, M-CSF, HGF, MIF, PDGF-AA, and lipocalin-2 were increased in STK2 compared to DMEM/FBS. Factors related to cell migration, including EMMPRIN, G-CSF, CCL-7, CCL3, MIF, PDGF-AA, and MMP-9, were significantly increased in STK2 compared to DMEM/FBS. In addition, factors related to anti-inflammation, including GDF-15, ICAM-1, IL-1ra, CCL-20, TFF-3, and thrombospondin-1 were significantly increased in STK2 compared to DMEM/FBS. Furthermore, factors related to stemness/differentiation, including CD105, M-CSF, IGFBP-2, PDGF-AA, CXCL-4, thrombospondin-1, and uPAR, were significantly increased in STK2 compared to DMEM/FBS. Adiponectin and FGF-19, factors related to metabolism, were also increased in STK2 compared to DMEM/FBS. Cell surface CD105 expression of ASCs decreased when cultured in STK2, as shown in Figure 2. However, interestingly, soluble CD105/endoglin (sENG) expression increased in the culture supernatant of ASCs using STK2 compared to DMEM/FBS (Figure 8B). It is known that sENG antagonizes the binding of TGF-β1 to its receptor and its downstream signaling [44]. These results suggest that ASCs cultured using STK2 have an advantage in treatment of diseases related to TGF-β1, including lung fibrosis and liver cirrhosis.

## 3. Discussion

FBS provides a variety of elements required for cell growth, including growth factors, vitamins, amino acids, carbohydrates, iron transporters, and hormones. However, whether the use of FBS in cell culture is the best choice has recently been questioned. Though FBS can induce the growth of a variety of cell types, the mechanism by which it does so and the specific factors that are critical for individual cell types have not been identified yet. FBS is harvested from cows, and is affected by both genes and environment. In terms of genetic background, there are no homogenized cow lines, unlike mice bred specifically for laboratory use. Although cows have identical genetic backgrounds, the environment, including microbes and diet, to which they are exposed can differ. This has the potential to affect the health of the animal and, consequently, specific factors in the blood. Since diet is not regulated among all cows, the levels of a variety of factors, including amino acid, growth factors, fats, and carbohydrate in the blood show drastic variation from cow to cow. In addition, the level of attachment factors in the blood has a variety of effects on different cell types. For instance, binding to attachment factors can initiate an inflammatory response in antigen-presenting cells in the immune system such as macrophages and dendritic cells [45,46]. In addition, attachment factors such as fibrinogen and vitronectin can induce differentiation of stem cells [47,48].

Unlike laboratory mice held in tightly controlled facilities, cows are exposed to the environment and environmental pathogens. Most pathogens are directly transferred from mother to fetus through the blood. The most common pathogens in serum are prions, the causative agent of mad cow disease that causes plaques in the brain and leads to neurological malfunction. Since prions are an altered version of endogenous proteins, it is not easy to test or examine the presence of prions in serum. In addition, cows are also exposed to various viruses, including bovine adenovirus, bovine parovirus, reovirus, parainfluenza-3 virus, rabies virus, and bluetongue virus, which are currently examined. However, unknown viruses are still infested in the blood of cows. Therefore, the existence of virus in serum should be further tested, which leads to the complex process of manufacturing and price rise of FBS.

Based on the variable and uncontrolled nature of FBS, the use of serum in the development of cell therapeutics may be questioned. First, the use of serum presents obstacles in creating a reproducible end product. Second, it is hard to supply FBS originated from a single lot and in sufficient quantities. In this study, we have shown that culture using serum-free chemically-defined media has many advantages for therapeutic application of MSCs compared with conventional medium containing serum. The results of our analysis would help to introduce serum-free, chemically-defined media for clinical application and preclinical research.

## 4. Materials and Methods

### 4.1. Preparation of Adipose-Derived Mesenchymal Stem Cells

Human subcutaneous fat tissue was isolated via liposuction after informed consent was obtained (Abion Inc., Seoul, Korea, IRB approval number 198034-161018-BR-001-02), and adipocyte-derived MSCs were isolated by digestion of 0.075% type I collagenase as described previously [49]. ASCs were cultured in low glucose DMEM supplemented with 10% FBS and 1% penicillin/streptomycin, or ASCs were established in STK1 medium (Abion Inc., Seoul, Korea). In the case of STK1, cells were washed twice at 4 days after seeding. After reaching 85% confluency, cells were digested with 0.05% TE and seeded (3 × 10^5^ cells) into 100 mm dish (Corning, Midland, NC, USA) containing STK2 (Abion Inc., Seoul, Korea).

### 4.2. Analysis of Population Doubling Time and Cell Viability

ASCs (3 × 10^4^ cells) at passage (P) 2 were seeded onto 6-well plates (Corning, Midland, NC, USA) and cultured at 37 °C in a humidified 5% CO_2_ incubator. When the cells reached 90% confluency, cells were detached with 0.05% TE, and subcultured again to P15. At each subculture, numbers of harvested cells were counted using a hemocytometer. PDT and accumulated cell numbers were deduced by the following equation: $T\times \frac{\log\left(2\right)}{\log\left(q2\right)-\log\left(q1\right)}$; T, cell culture time; q1, initial number of cells; q2, final number of cells. For cell viability assay, cells were stained with 0.4% trypan blue and live cells were counted using Cedex Cell Counter (Roche, Branchburg, NJ, USA).

### 4.3. Flow Cytometry

To examine the expression of ASC markers, cells (1 × 10^5^ cells) were incubated with anti-CD29-PE, anti-CD44-PE, anti-CD105-PE, anti-CD34-FITC, anti-CD45-PE, and anti-HLA-DR antibodies for 2 h at 4 °C (BioLegend, San Diego, CA, USA). Antibody-positive populations were quantitated using FACS-Calibur (BD Biosciences, San Jose, CA, USA) and analyzed using FlowJo (Tree Star, Ashland, OR, USA). Stain Index (SI) values for each (+) and (−) MSC biomarker were calculated using the following formula: $\mathrm{SI}=\frac{\mathrm{MFI}1-\mathrm{MFI}2}{2\times \mathrm{SD}}$; MFI1, geometric mean fluorescence of control stain; MFI2, geometric mean fluorescence of test stain; SD, standard deviation of geometric mean fluorescence.

### 4.4. Differentiation Analysis

To induce adipogenic differentiation, ASCs were cultured to confluency and then further cultured for 2 days. Then, media was changed to adipogenic medium (StemPro Adipogenesis Differentiation Kit, Gibco, Waltham, MA, USA) and adipogenesis was induced for 14 days. Cells were fixed with 10% formaldehyde for 15 min at room temperature (R.T.), and washed with 60% isopropanol. Cells were stained with 0.6% Oil Red O solution for 45 min at R.T. Cells were washed with 60% isopropanol and distilled water (D.W.). To induce osteogenic differentiation, ASCs were cultured to confluency and then further cultured for 2 days. Then, media was changed to DMEM containing 10% FBS, 1% GlutaMAX, 0.2 mM ascorbic acid, 10 mM glycerol 2-phosphate, 1% penicillin/streptomycin, and 0.1 μM dexamethasone and cultured for 21 days. Cells were fixed as described above and washed three times with D.W. Then, cells were stained with 2% Alizarin Red S solution and washed again with D.W. To induce chondrogenic differentiation, cells were cultured in DMEM containing 10% FBS, 1% penicillin/streptomycin, 1% insulin-transferrin-selenium-A supplement, 50 μM ascorbic acid, 100 nM dexamethasone, and 10 ng/mL transforming growth factor-β (TGF-β) for 21 days. Cells were fixed as described above and stained with 0.5% Alcian blue. Differentiation was quantitated and evaluated by ImageJ (NIH, Bethesda, MD, USA), and expressed as an arbitrary intensity.

### 4.5. Cytokine Proteome Array

To analyze the secretome of ASCs that were cultured in DMEM containing 10% FBS or STK2, culture media was harvested at P5 and P10. Relative cytokine expression was quantitated using a cytokine array kit (R & D Systems, Minneapolis, MN, USA) following the manufacturer’s protocol. Dot intensity was analyzed using ImageJ.

### 4.6. Analysis of Attached Cell Size

ASCs were seeded onto 24-well plates and cultured for 24 h. Morphology of ASCs was observed in 100× magnification images that were automatically taken using IncuCyte ZOOM Live-Cell Analysis System (Essen Bioscience, Ann Arbor, MI, USA). Images were then analyzed for adherent ASC size using ImageJ 1.50i (NIH, USA).

### 4.7. Cytokinesis-Block Micronucleus Assay

Cells were seeded onto 60 mm culture dishes (Corning Inc., Corning, NJ, USA) and incubated at 37 °C and 5% CO_2_ overnight. After cell confluency reached about 60%, the positive control group was treated with 0.25 μg/mL of mitomycin C (mit-C; Sigma, San Jose, CA, USA) dissolved in fresh medium for 24 h, whereas the negative group received a medium change. After 24 h, the culture medium was replaced with fresh culture medium with 1.5 μg/mL cytochalasin B (cyt-B; Sigma, San Jose, CA, USA). Cells were treated with cyt-B for 24 h and collected by trypsinization and centrifuged gently at 400× *g* for 5 min at R.T. Cells were gently resuspended by tapping and were exposed to hypotonic solution as previously described [41]. The cell pellet was gently resuspended in 150–200 μL of fresh fixative solution. A glass slide was placed over a warmed (42 °C) water bath or heat block. An aliquot of 20–25 μL of the cell suspension was distributed onto the glass slide over heat for about 10–15 s and then left to air-dry. Slides were incubated for 30 min at 60 °C and then stained with 10% Giemsa stain (Sigma, San Jose, CA, USA). Slides were prepared in triplicate from each culture and 100 binucleated cells were analyzed for each replicate, for a total of 300 cells in each experimental group. The binucleated cells were scored for the presence of MN, NPBs, and NBUDs at 200× and 400× magnifications using Eclipse 80i (Nikon, Tokyo, Japan).

### 4.8. Quantitative Reverse-Transcription (RT)-PCR

Total RNA was isolated from ASCs at P5 and P10 using an RNA extraction kit (Hybrid R, GenAll, Seoul, Korea). cDNA was synthesized by reverse transcription with 2 μg of total RNA using a cDNA synthesis kit (Roche, Branchburg, NJ, USA), and then each gene transcript was amplified by quantitative RT-PCR with specific primers. The primers used are summarized in Table 3.

### 4.9. β-Galactosidase Staining

For cellular senescence analysis, MSCs (3 × 10^4^) were seeded onto 6-well plates and cultured to 80% confluency in DMEM containing 10% FBS or STK2. Then, cells were washed with PBS and fixed with PBS containing 2% paraformaldehyde and 0.2% glutaraldehyde for 5 min at R.T. Cells were washed twice with PBS, and stained with staining solution containing 40 mM citric acid, 5 mM K_4_[Fe(CN)_6_]∙3H_2_O, 5 mM K_3_[Fe(CN)_6_], 150 mM NaCl, 2 mM MgCl_2_, and 1 mg/mL X-gal at 37 °C for 12 h [50]. Cells were washed twice with PBS, once with methanol, and allowed to air dry. The β-galactosidase-positive cells were captured under bright-field microscopy (Nikon, Japan).

### 4.10. Statistical Analyses

The data are expressed as mean ± SD, mean ± SEM, or median ± SD. The results were analyzed using GraphPad Prism, version 7.00 for Windows (GraphPad Software, La Jolla, CA, USA). Student’s *t*-tests were performed to determine the statistical significance of group differences and a *p*-value < 0.05 was considered significant.

## Figures and Tables

**Table 1:** Stain Index (SI) values of FACS analysis for detection of positive and negative MSC biomarker.

Antibody	Passage	DMEM/FBS	STK2
CD29	P3	30.5 ± 5.1	57.0 ± 13.7
	P9	19.8 ± 7.4	48.4 ± 15.6
CD44	P3	26.6 ± 7.4	39.9 ± 5.42
	P9	16.6 ± 3.7	37.5 ± 0.88
CD73	P3	27.7 ± 5.5	41.5 ± 6.31
	P9	32.5 ± 6.5	43.5 ± 7.35
CD90	P3	31.8 ± 6.1	38.3 ± 2.91
	P9	28.9 ± 5.3	46.3 ± 11.8
CD105	P3	32.7 ± 15.4	6.51 ± 5.39
	P9	22.3 ± 11.9	8.04 ± 0.46
CD34	P3	0.09 ± 0.09	0.19 ± 0.03
	P9	0.26 ± 0.11	0.14 ± 0.17
CD45	P3	0.05 ± 0.01	0.08 ± 0.09
	P9	0.01 ± 0.02	0.13 ± 0.06
HLA-DR	P3	−0.03 ± 0.01	−0.01 ± 0.01
	P9	−0.06 ± 0.09	−0.04 ± 0.02

*n* = 3; mean ± SD.

**Table 2:** Up- or down-regulated factors produced by ASCs cultured in STK2.

	Name	Function
Up	Adiponectin	regulate glucose levels, fatty acid breakdown, adipocyte differentiation
	Angiogenin	induce angiogenesis
	Cystatin C	induce proliferation of neural stem cell
	DKK_1	inhibit WNT signaling pathway
	EMMPRIN	induce matrix metalloproteinase synthesis
	CD105	mediate TGFβ signal
	FGF-19	regulate cell proliferation, glucose and bile acid metabolism
	G-CSF	induce mesenchymal cell mobilization
	GDF-15	regulate inflammatory and apoptotic pathways in injured tissues
	GM-CSF	promote proliferation of human fetal and adult microglia
	M-CSF	regulate the survival, proliferation and differentiation of hematopoietic precursor cells
	HGF	promote progenitor cell mobilization, induce angiogenesis and cell proliferation, inhibit immune cell proliferation
	CD54	induce MSC-mediated immunosuppression
	IGFBP-2	promote cell mobility and osteoblast differentiation
	IL-1ra	inhibit the pro-inflammatory effect of IL-1β
	Lipocalin-2	protect MSC against unfavorable microenvironments and decrease MSC senescence
	CCL-7/MCP-3	induce MSC migration and recruit MSC to site of injured tissue
	MIF	Induce MSC migration and promote cell proliferation of neural stem/progenitor cell
	CCL3	induce MSC migration and recruit MSC to site of injured tissue
	CCL-20	inhibit T cell activation and proliferation and induce MSC migration
	PDGF-AA	induce MSC migration and promote osteogenic differentiation
	MMP-9	ECM remodeling and cell migration
	TSF-14	inhibit atherosclerosis and protect cardiac function
	CXCL4	protect MSC from acute radiation injury and maintain HSC stemness
	TFF3	decrease IL-6 and IL-8
	Thrombospondin-1	induce MSC differentiation
	uPAR	inhibit tumor growth and induce chemotaxis of CD34^+^ hematopoietic stem cell
Down	FGF-7	reduce IL-1β and TNFα, and increase IL-10
	IGFBP-3	induce cellular senescence
	IL-11	induce inflammation to cancer progression and function as tumor promoting cytokine

**Table 3:** Primer sequences used for qRT-PCR.

Primers	Sequences
CD29	F, 5′-cgatgccatcatgcaagt-3′
	R, 5′-acaccagcagccgtgtaac-3′
CD44	F, 5′-tgcctttgatggaccaatta-3′
	R, 5′-ggggtgtacagtagaaaagg-3′
CD73	F, 5′-ccagtccactggagagttcc-3′
	R, 5′-cgacacttggtgcaaagaac-3′
CD90	F, 5′-cagaacgtcacagtgctcaga-3′
	R, 5′-gaggagggagagggagagc-3′
CD105	F, 5′-acgctccctctggctgtt-3′
	R, 5′-gctgaaggtcacaatggactg-3′
PPARγ	F, 5′-tcgctgatgcactgcctatg-3′
	R, 5′-gagaggtccacagagctga-3′
Aggrecan	F, 5′-gtggagccgtgtttccaag-3′
	R, 5′-agatgctgttgactcgaacct-3′
Osteocalcin	F, 5′-cgctaccttggagcttcagt-3′
	R, 5′-gtttggctttagggcagcac-3′
GAPDH	F, 5′-accccagcaaggacactgagcaag-3′
	R, 5′-ggctccctaggcccctcctgttatt-3′

## Abbreviations

MSC	Mesenchymal stem cell
ASC	Adipose-derived stem cell
FBS	Fetal bovine serum
PDT	Population doubling time
SSC	Side scatter channel
CBMN	Cytokinesis-block micronucleus
NPB	Nucleoplasmic bridges
NBUD	Nuclear buds
